# Enhanced Acetone Sensing Properties Based on Au-Pd Decorated ZnO Nanorod Gas Sensor

**DOI:** 10.3390/s24072110

**Published:** 2024-03-26

**Authors:** Yinfeng Shen, Yiping Liu, Chao Fan, Qudong Wang, Ming Li, Zhi Yang, Liming Gao

**Affiliations:** 1 State Key Laboratory of Metal Matrix Composites, School of Material Science and Engineering, Shanghai Jiao Tong University, Shanghai 200240, China; syf1999@sjtu.edu.cn (Y.S.); liuyp37@sjtu.edu.cn (Y.L.); mingli90@sjtu.edu.cn (M.L.); 2 Key Laboratory of Thin Film and Microfabrication (Ministry of Education), Department of Micro/Nano Electronics, School of Electronic Information and Electrical Engineering, Shanghai Jiao Tong University, Shanghai 200240, China; chaofan@sjtu.edu.cn; 3 National Engineering Research Center of Light Alloy Net Forming, State Key Laboratory of Metal Matrix Composites, School of Materials Science and Engineering, Shanghai Jiao Tong University, Shanghai 200240, China; wangqudong@sjtu.edu.cn

**Keywords:** gas sensor, acetone, ZnO nanorod, Au-Pd nanoparticle

## Abstract

The mature processes of metal oxide semiconductors (MOS) have attracted considerable interest. However, the low sensitivity of metal oxide semiconductor gas sensors is still challenging, and constrains its practical applications. Bimetallic nanoparticles are of interest owing to their excellent catalytic properties. This excellent feature of bimetallic nanoparticles can solve the problems existing in MOS gas sensors, such as the low response, high operating temperature and slow response time. To enhance acetone sensing performance, we successfully synthesized Au-Pd/ZnO nanorods. In this work, we discovered that Au-Pd nanoparticles modified on ZnO nanorods can remarkably enhance sensor response. The Au-Pd/ZnO gas sensor has long-term stability and an excellent response/recovery process. This excellent sensing performance is attributed to the synergistic catalytic effect of bimetallic AuPd nanoparticles. Moreover, the electronic and chemical sensitization of noble metals also makes a great contribution. This work presents a simple method for preparing Au-Pd/ZnO nanorods and provides a new solution for the detection of acetone based on metal oxide semiconductor.

## 1. Introduction

Volatile organic compounds are common pollutants in air. Many VOCs are known to be toxic and even considered carcinogenic. Acetone (CH_3_COCH_3_) is a typical volatile organic compound and is widely applied in various fields, like the medicine, agriculture and chemical industries, etc. [1]. However, acetone is dangerous, toxic and detrimental to human beings. The lower and upper explosive limits of acetone are 2.6% and 12.8%, making it a flammable gas with relatively active chemical properties [2]. The human body’s organs can be seriously damaged when exposed to acetone above 173 ppm for a long time. In order to protect human health, the threshold for people’s exposure to acetone at work is set at 250 ppm [3]. Therefore, there is a great need to invent a relatively high-sensitivity acetone gas sensor.

Over the last couple of years, many gas sensors have been employed due to the requirement for environmental gas detection. Metal oxide semiconductors have received significant interest because of their mature process, chemical stability and low cost [4]. Among them, gas sensors made of SnO_2_ [5], WO_3_ [6], ZnO [7], In_2_O_3_ [8,9] and other materials have been widely reported for acetone detection. Zinc oxide (ZnO) is considered a crucial material for n-type semiconductors due to its exceptional thermal stability, wide energy bandgap and high electron mobility [10]. Compared to other metal oxides, ZnO is relatively low-cost, easy to produce in large quantities and non-toxic [11]. Zinc oxide also allows for the detection of oxidizing and reducing gases over a wide detection range with high response [12]. However, the poor selectivity and insensitive response of pure ZnO gas sensors have become urgent problems to be solved. ZnO are synthesized with various morphologies, such as nanowires [13,14], nanosheets [15,16] and nanospheres [17,18]. These types of morphologies have a large surface area, allowing for full contact with the specific gas. Their complex morphology can also provide more active sites, enhancing the reaction efficiency between the target gas and the material. Nevertheless, the construction of heterostructures [19,20], noble metal surface modification such as Au [21], Pt [22] and Pd [23] and ultraviolet irradiation [24,25] have been shown to be viable methods of improving ZnO’s sensing performance. Noble metal modification has attracted considerable attention due to its mature preparation process and significant improvement in the performance of gas sensing.

It is widely acknowledged that the noble metal modification method is effective because of the impacts of chemical and electronic sensitization [26]. Noble metals function as catalysts for the surface reactions of materials and serve as sites for oxygen adsorption. For example, Wang [27] et al. fabricated ZnO flowers using thermal decomposition in organic solvents, and then, Au was loaded onto ZnO. The ZnO gas sensor modified by gold nanoparticles had better acetone-sensing performance. Song [28] et al. prepared Pt-In_2_O_3_ nanotubes, which have increased sensitivity and selectivity and a reduced optimal operating temperature compared to pure In_2_O_3_ nanotubes. Compared with single metal modification, the catalytic performance of noble metals can be enhanced through bimetallic modification by transferring electrons between two metals. Moreover, a bimetal can further enhance the response and shorten the response time of a single metal gas sensor. The synergistic effect produced by this process has received widespread attention in gas sensors. For example, Liu [29] et al. modified a SnO_2_ surface with a AuPd alloy, which had a higher response and selectivity than the pure SnO_2_ material. Wang [30] et al. proposed PtAu alloy modified flower-like WO_3_ for n-pentanol gas sensing. The response was 105 times that of pure WO_3_. High-response and -sensitivity gas-sensing materials can be prepared by modifying metal oxide semiconductors with bimetallic materials. Hence, we utilize the synergistic effect produced by the distinct work functions of Pd and Au to enhance the gas detection capabilities of ZnO.

In this study, we used a simple hydrothermal technique to synthesize ZnO nanorods and modified ZnO nanorods with Au and Pd nanoparticles. According to the tests, the Au-Pd/ZnO gas sensor is more sensitive to acetone. Moreover, the Au-Pd/ZnO gas sensor has a quicker response/recovery process, large detection range and excellent long-term stability. In summary, ZnO nanorods shows an excellent response to acetone after modifying Au and Pd nanoparticles. The Au-Pd/ZnO gas sensor has great application potential for acetone detection.

## 2. Materials and Methods

### 2.1. Preparation of ZnO Nanorods

Sodium hydroxide (NaOH) and sodium borohydride (NaBH_4_) were purchased from Sinopharm Chemical Reagent Co., Ltd. (Shanghai, China). Zinc acetate dihydrate (Zn(CH_3_COO)_2_·2H_2_O) was purchased from Shanghai Aladdin Biochemical Technology Co., Ltd. (Shanghai, China). We purchased all reagents without further purification from specific suppliers. Typically, 0.55 g zinc acetate dihydrate (Zn(CH_3_COO)_2_·2H_2_O) was added to 25 mL deionized water and stirred for 5 min. A uniform suspension was formed by adding 1 g NaOH to the above solution and stirring continuously for 15 min. Finally, a Teflon-lined stainless-steel autoclave was used to maintain the suspension at 200 °C for 24 h. The hydrothermal sample powder was then naturally cooled to the environmental temperature and centrifuged several times with absolute ethanol and deionized water. Finally, the powder was dried at 60 °C for 24 h.

### 2.2. Preparation of Au/ZnO, Pd/ZnO and Au-Pd/ZnO

The Au-Pd/ZnO nanorods were prepared as follows. A total of 15 mL deionized water containing 50 mg ZnO nanorod powder was sonicated for 10 min. Subsequently, 394 μL H_2_PdCl_4_ (20 mM) along with 213 μL HAuCl_4_ (20 mM) was added to the above solution. Once the mixture achieved a uniform distribution, the above solution was stirred for 4 h with 3 mL of prepared 100 mM NaBH_4_ solution. Water and absolute ethanol were used to wash the precipitate multiple times. In the final step, the resultant sample was dried at 60 °C for 24 h.

**Figure 1:**
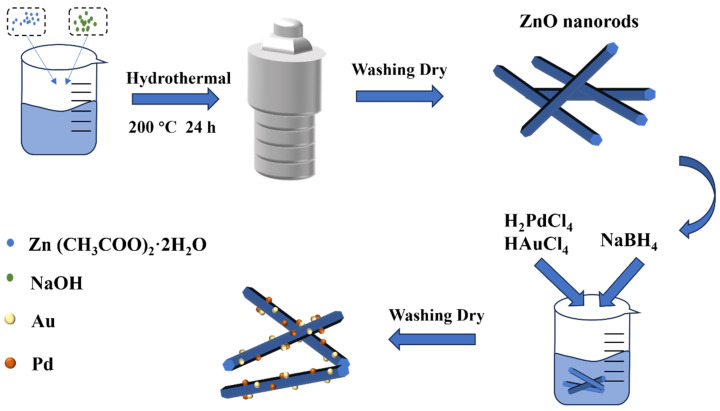
Schematic diagram of the synthesis of Au-Pd-decorated ZnO nanorods.

Similarly, the synthesis of Au/ZnO and Pd/ZnO nanorods only required the introduction of 426 μL HAuCl_4_ (20 mM) or 788 μL H_2_PdCl_4_ (20 mM) into the respective solution. The remaining preparation techniques aligned with the synthesis approach employed for Au-Pd/ZnO nanorods (Figure 1).

### 2.3. Characterization

We examined the samples using X-ray diffraction (XRD, D8 Advance, Karlsruhe, Germany). Field emission scanning electron microscopy (SEM, Zeiss GeminiSEM 300, Oberkochen, Germany) and transmission electron microscopy (TEM, JEOL-2100F, Tokyo, Japan) were used to observe the morphology and microstructure of the samples. Analysis of surface elements was conducted using X-ray photoelectron spectroscopy (XPS, Esca Lab 250Xi, Waltham, MA, USA).

### 2.4. Fabrication and Performance Test of Gas Sensor

To fabricate the gas sensor, the sensing material was first produced by mixing a certain amount of powder with absolute ethanol. A certain amount of sensing material was covered to an alumina tube and subsequently dried at a temperature of 60 °C for 2 h. For controlling temperature changes, we inserted a heater produced by a Ni-Cr alloy coil into the alumina tube. This article used the JF-02F gas sensor test system (Gui Yan Jin Feng Tech. Co., Ltd., Yunnan, China) to test the sensors’ gas sensing capabilities. The gas was obtained by evaporating the organic solution on the evaporator with the help of a micro-syringe in the testing chamber. The target gas was discharged once the sensor’s resistance reached a relative steady value. The liquid volume of the target gas was calculated according to the following formula:

(1) $$V=\frac{C\times M\times V_{m}}{\rho \times R\times T}$$

where V is the volume of injected liquid, C is the required vapor volume fraction, M is the molecular weight of the liquid, *V*_m_ is the volume of the testing chamber (18.5 L), ρ is the density of the liquid, R is the gas constant (0.08206 L atm/mol K), and T is the ambient temperature. A gas sensitivity test was performed at a relative humidity of ~35% RH. We used R = R_a_/R_g_ to determine the sensor’s response. In specific gas or air, the sensor’s resistance is represented as R_g_ or R_a_. During target gas introduction or exhaustion, the time required to achieve a 90% change in sensor resistance is called the response and recovery time.

## 3. Morphology and Structural Characterization

**Figure 2:**
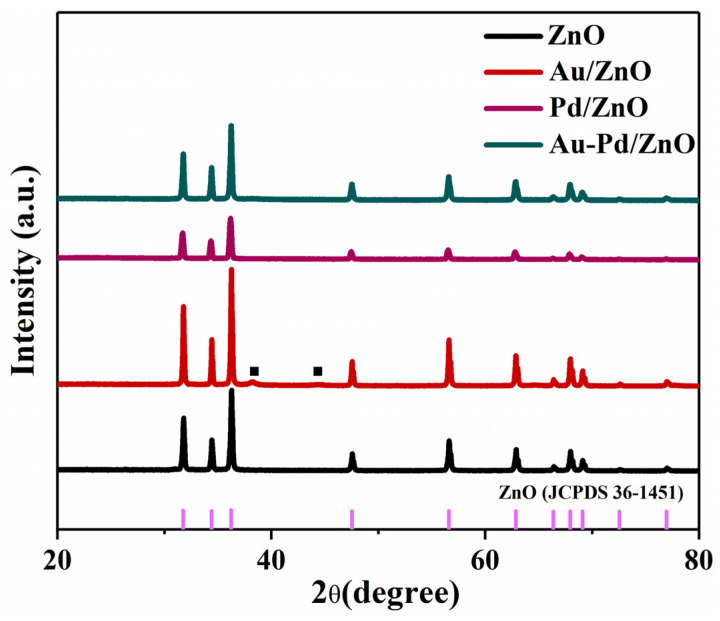
XRD spectra of ZnO, Au/ZnO, Pd/ZnO and Au-Pd/ZnO.

The structure of ZnO and ZnO modified with Au, Pd and Au-Pd nanoparticles was investigated using XRD (Figure 2). Each sample has a strong diffraction peak consistent with ZnO’s hexagonal structure (JCPDS: 36-1451). None of additional impurity peaks are detected in the four samples, suggesting that nanoparticles of Au and Pd do not alter the lattice structure of ZnO nanomaterials. Two weak diffraction peaks can be observed at 38.2° and 44.4° for the Au/ZnO sample, indicating the presence of (111) and (200) of the face-centered cubic Au (JCPDS: 04-0784). The XRD’s limited sensitivity results in the observation of no other diffraction peaks for the Pd/ZnO and Au-Pd/ZnO samples.

**Figure 3:**
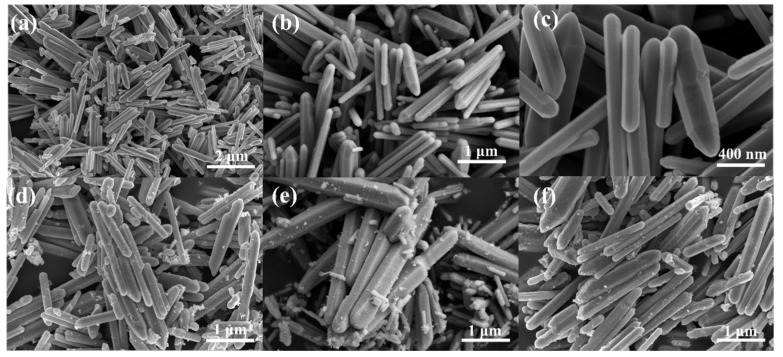
SEM images of (**a**–**c**) ZnO; (**d**) Au-ZnO; (**e**) Pd/ZnO; and (**f**) Au-Pd/ZnO.

An exploration of the morphology of pristine ZnO and ZnO modified with Au, Pd and Au-Pd nanoparticles was performed using scanning electron microscopy (Figure 3). All samples show one-dimensional nanorod-like structures. The lengths are concentrated in the range of 1–3 μm and the morphologies present a disorderly stacked structure. The pure ZnO nanorods are smooth (Figure 3a–c). The one-dimensional structures possess a high surface area, which facilitates the diffusion of gas molecules. It is evident from Figure 3d–f that either Au or Pd nanoparticles exhibit a uniform distribution upon the smooth ZnO surface, indicating that the metal nanoparticles are successfully modified on ZnO nanorods. The presence of noble metal (Au, Pd and AuPd) on the surface of ZnO nanowires can effectively increase the contact area of the material with the gas. Furthermore, the presence of noble metals can also enhance the reaction sites of ZnO with acetone gas, thereby accelerating the reaction rate and increasing the response to the target gas [31,32]. The morphology of ZnO nanorods remains unchanged by the addition of Au and Pd nanoparticles. 

**Figure 4:**
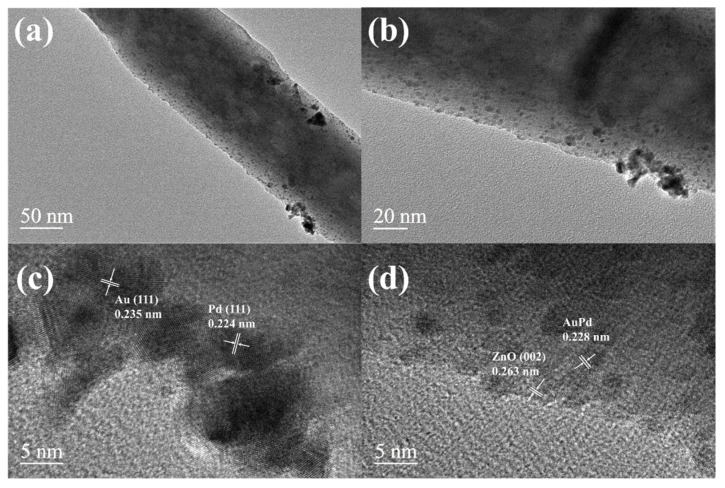
(**a**,**b**) TEM images and (**c**,**d**) HRTEM images of Au-Pd/ZnO.

**Figure 5:**
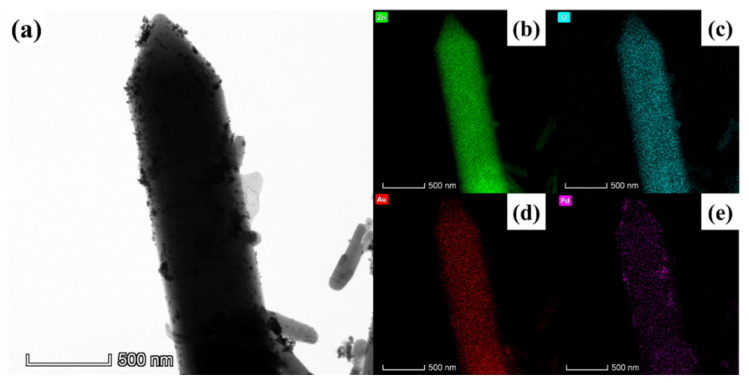
(**a**) TEM image and mapping: (**b**) Zn; (**c**) O; (**d**) Au; and (**e**) Pd images of Au-Pd/ZnO.

The analysis of Au-Pd/ZnO’s morphology and microstructure was conducted using TEM and HRTEM. As shown in the low-magnification TEM (Figure 4a,b), the surface of ZnO is evenly covered with numerous ~5 nm noble metal nanoparticles. The fringe spacing values of 0.224 and 0.235 nm correspond to the lattice plane of Pd (111) and Au (111), respectively. The lattice distance results indicate that Pd and Au particles coexist on pure ZnO. In Figure 4d, in addition to the 0.263 nm lattice spacing corresponding to ZnO (002), there are also 0.228 nm lattice stripes. This 0.228 nm lattice spacing is between Au (111) and Pd (111), which indicates the possible existence of a AuPd alloy [29]. Element analysis using mapping (Figure 5) also confirms the existence of Zn, O, Au and Pd.

**Figure 6:**
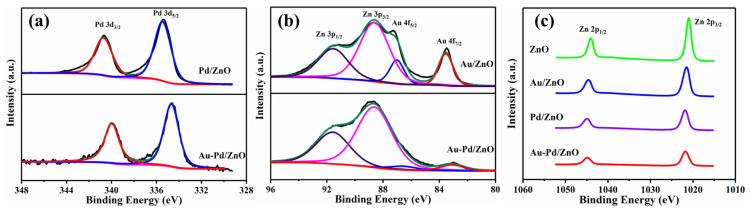
The high-resolution XPS spectra of (**a**) Pd 3d; (**b**) Au 4f; and (**c**) Zn 2p.

The elemental composition of pure ZnO and composite materials of ZnO with Au, Pd and Au-Pd modification was further determined using XPS analysis. The elemental analysis of the energy spectrum (Figure 6) further proved the existence of Zn, Au and Pd, where Zn elements are derived from ZnO. Pd and Au elements are derived from the modified noble metal nanoparticles Au and Pd. Figure 6a shows the Pd 3d spectra of both Pd/ZnO and Au-Pd/ZnO composite materials. The binding energy peaks observed at ~340.7 eV and ~335.5 eV belong to Pd 3d_3/2_ and Pd 3d_5/2_ in Pd/ZnO [33]. The peaks detected in the Au/ZnO sample (Figure 6b) at ~83.6 eV and ~87.0 eV accord with Au 4f_7/2_ and Au 4f_5/2_ [34]. It may be beneficial for the interaction between Pd and Au that the Pd 3d and Au 4f peak positions in Au-Pd/ZnO shift towards lower energies compared to Pd/ZnO and Au/ZnO [35]. Figure 6c displays the X-ray photoelectron spectra (XPS) of Zn 2p for the four samples. Zn 2p_3/2_ and Zn 2p_1/2_ accord with the binding energies at ~1021.0 eV and ~1044.1 eV of ZnO. Compared with pure ZnO, the Zn 2p peaks of the Au-Pd/ZnO, Au/ZnO and Pd/ZnO composite materials shift towards higher binding energy. The higher work function of noble metal Au and Pd nanoparticles compared to ZnO may lead to electron transfer from ZnO to noble metals [36]. 

## 4. Gas-Sensing Performance

**Figure 7:**
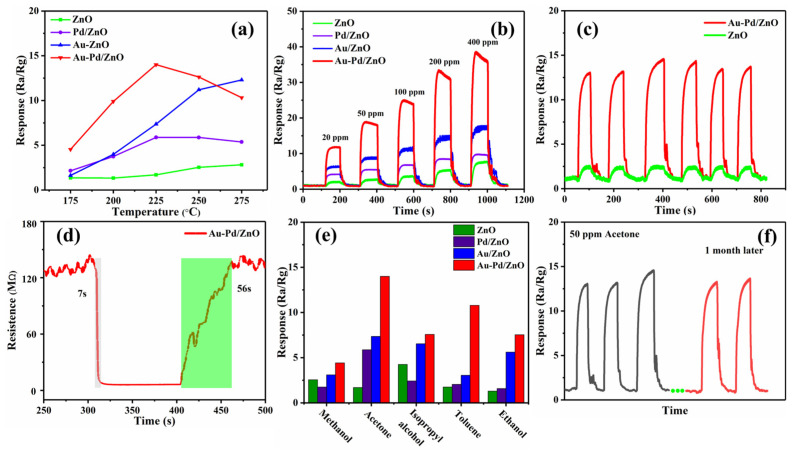
Gas-ensing characteristics of prepared samples: (**a**) response of four samples to 50 ppm acetone at different temperatures; (**b**) dynamic response curve of sensors to 20–400 ppm acetone at 225 °C; (**c**) response curve of ZnO and Au-Pd/ZnO to 50 ppm acetone at 225 °C; (**d**) response/recovery time of Au-Pd/ZnO to 50 ppm acetone at 225 °C; (**e**) responses based on four gas sensors to 50 ppm of different target gases at 225 °C; (**f**) long-term stability of Au-Pd/ZnO after one month.

The gas sensors were tested using JF-02F. An acetone gas concentration of 50 ppm was applied to the four sensors (ZnO, Pd/ZnO, Au/ZnO and Au-Pd/ZnO) at a range from 175 to 275 °C (Figure 7a). The Au-Pd/ZnO gas sensor shows an initial increase followed by a subsequent decrease in response to temperature changes. Under 225 °C, the Au-Pd/ZnO gas sensor exhibits the maximum response. Therefore, the Au-Pd/ZnO gas sensor can operate at 225 °C efficiently. Before 225 °C, the adsorption rate increases as the temperature increases, so the sensor response increases simultaneously. When the temperature is beyond 225 °C, it is too high and not conducive to the adsorption of gas molecules on ZnO. With 50 ppm acetone gas, the respective responses of ZnO, Pd/ZnO and Au/ZnO are ~2.2, ~5.9 and ~7.4 at 225 °C. The response of ZnO towards acetone gas can be enhanced through the introduction of a noble metal like Au, Pd or AuPd due to the electronic and chemical sensitization of Au and Pd and the synergistic effect between Au and Pd.

Figure 7b illustrates the dynamic responses of composite materials of pure ZnO and ZnO with Au, Pd and Au-Pd modification to acetone gas ranging from 20 to 400 ppm at 225 °C. Under a fixed gas concentration, the response always maintains the relationship of Au-Pd/ZnO > Au/ZnO > Pd/ZnO > ZnO. Because acetone concentration exceeding 173 ppm will seriously affect the central nervous system [3], the Au-Pd/ZnO gas sensor can meet the detection requirements very well. Furthermore, the Au-Pd/ZnO gas sensor demonstrates not only a higher response, but also the capability of a rapid response and recovery (7 s and 56 s). It should be noted that the recovery process can be accelerated by rationally controlling the bimetallic content [37]. The synergistic catalytic effect of AuPd bimetallic nanoparticles can explain this quick response/recovery speed. The collaboration of Pd and Au produces a synergistic catalytic effect that can accelerate the absorption and dissociation of oxygen and reduce the reaction’s activation energy, which accelerates the interaction between acetone and adsorbed oxygen ions [38]. 

The cyclic repeatability and selectivity of gas sensors in complex gas situations are also important parameters for gas sensors. Figure 7c displays six response/recovery cycles of Au-Pd/ZnO and ZnO gas sensors towards 50 ppm acetone gas and air at 225 °C. Throughout the six response/recovery processes, the responses of the two sensors are minimally altered, while the Au-Pd/ZnO gas sensor shows a remarkably rapid response and recovery speed. This phenomenon indicates that the Au-Pd/ZnO and ZnO gas sensors have excellent repeatability. Moreover, the Au-Pd/ZnO gas sensor also exhibits good cycling characteristics at different temperatures (Figure S1). In daily applications, the composition of gases is very complex. The target gas is mixed among different gases in the atmosphere. Therefore, we compare the responses of the four sensors in 50 ppm methanol, acetone, isopropyl alcohol, toluene and ethanol at 225 °C (Figure 7e). In comparison to the other four gas sensors, the Au-Pd/ZnO sensor exhibits the highest response in various gases and is most sensitive to acetone. This is mainly due to the electronic and chemical sensitization of the Au-Pd bimetal and its unique synergistic effect, which greatly enhances its catalytic ability [39]. Hence, the Au-Pd/ZnO sensor has good selectivity for acetone gas and has certain value in practical applications. Furthermore, we additionally examine the durability of the Au-Pd/ZnO gas sensor at 225 °C in response to 50 ppm acetone gas (Figure 7f). After one month, the Au-Pd/ZnO gas sensor only exhibits a little response change and still has good response/recovery performance, illustrating its excellent long-term stability. The Au-Pd/ZnO gas sensors are more sensitive at lower working temperatures and exhibit shorter response times compared to the other acetone sensors (Table 1).

## 5. Gas-Sensing Mechanism

**Figure 8:**
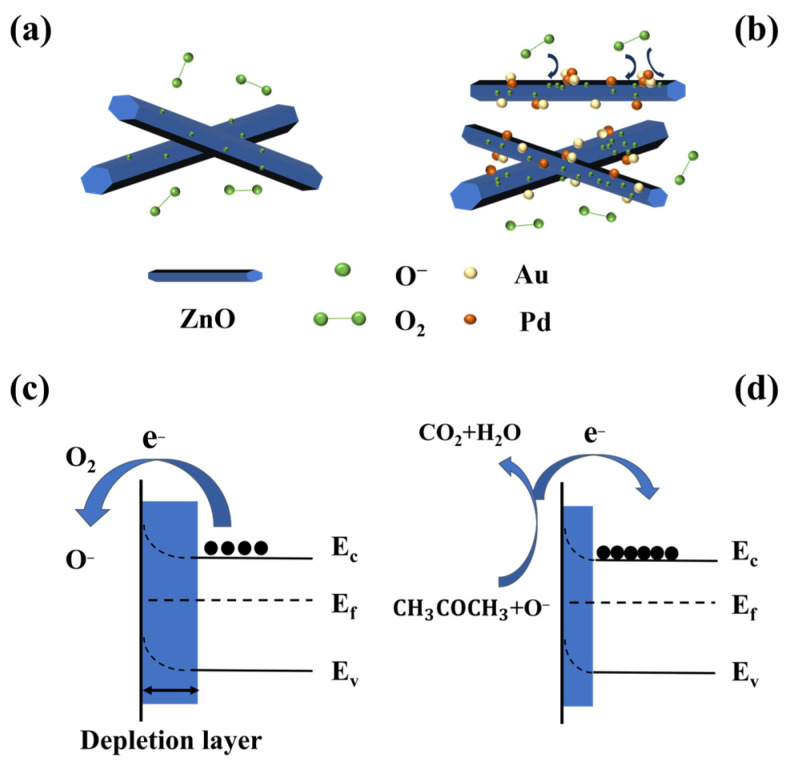
Schematic diagram of gas-sensing mechanism in air: (**a**) pure ZnO; (**b**) Au-Pd/ZnO sensors. Band diagram: (**c**) in air; (**d**) in acetone.

ZnO’s gas-sensing response primarily depends on changes in resistance resulting from the redistribution of electrons in various gas environments [46]. When zinc oxide is in contact with the air at the operating temperature, oxygen molecules capture electrons from the conduction band in ZnO to form chemically adsorbed oxygen ions (Figure 8c). The surface depletion layer of ZnO is successfully formed by the above process. The width of the depletion layer is closely related to conductivity. The thicker the depletion layer, the lower the electron density, resulting in higher resistance of ZnO [47]. The main types of adsorbed oxygen ions are determined by the gas sensor’s working temperature. Generally speaking, oxygen molecules capture electrons below 100 °C to form O_2_^−^ and O^−^ at 100–300 °C, and above 300 °C, O^2−^ is formed [48]. In this text, O^−^ is the primary type of adsorbed oxygen ion since the Au-Pd/ZnO gas sensor operates optimally at 225 °C. As shown below, the reaction equations are [49]

(2) $$O_{2\left(gas\right)}\to O_{2\left(ads\right)}$$

(3) $$O_{2\left(ads\right)}+e^{-}\to 2O_{\left(ads\right)}^{-}$$

When the sensor is exposed to reducing gases, chemisorbed oxygen is reduced by acetone gas. Electrons are released back into the conduction band of ZnO, ultimately leading to a narrow depletion layer and lower resistance (Figure 8d). 

The one-dimensional structure of ZnO nanorods, serving as a medium for electron transfer, exhibits a significant ratio of surface area to volume. Such a structure can increase the contact area with the target gas. Furthermore, the one-dimensional nanorod could offer an increased number of reaction sites for detecting acetone. This structure can facilitate the chemical adsorption and dissociation of oxygen, which can enhance gas-sensing capabilities (Figure 8a).

The presence of the Au and Pd noble metals and AuPd bimetal leads to better sensing capabilities of the Au-Pd/ZnO gas sensor compared to the pure ZnO gas sensor. First, as noble metals, Au and Pd have chemical sensitization effects (Figure 8b). Coating the ZnO surface with Au and Pd nanoparticles allows Au and Pd to convert more oxygen molecules into oxygen ions and overflow to the ZnO surface through their high catalytic activity or excellent “spillover effect”. A significant amount of oxygen ions can react with more acetone gas, which, in turn, enhances the gas response of the sensor. Simultaneously, the existence of Au and Pd can enhance the rate of electron transfer and significantly contribute to reducing the time required for response and recovery [31].

The exceptional sensing ability of the Au-Pd/ZnO gas sensor is also caused by electronic sensitization, since the work functions of ZnO, Au and Pd are 4.7 eV [50], 5.1 eV [51] and 5.12 eV [52], respectively. Because of the disparity in work functions, when Au, Pd and ZnO are in contact, electrons transfer from ZnO to Au or Pd. The process of electron flow is confirmed by XPS (Figure 6c). The movement of electrons will lead to the formation of the Schottky barrier, which will result in thickening of the electron depletion layer. Furthermore, this process can prevent the recombination of isolated electron–hole pairs, which helps to facilitate the transformation of electrons [53]. 

Differences in the work functions of Au and Pd noble metals lead to electron transfer between the two noble metals. This process will lead to a synergistic effect in the Au and Pd metals [54]. The adsorption and catalytic efficiency may be affected due to the introduction of another noble metal to a composite structure, which will alter the electronic or d-band configuration [55]. Compared with a single noble metal (Au or Pd), bimetal AuPd can dissociate more molecular oxygen into adsorbed oxygen ions [56]. The adsorbed oxygen ions can fully react with acetone gas, resulting in significant enhancement of the pure ZnO response towards acetone. Furthermore, the AuPd bimetal also induces more electron sensitization [32]. More electron sensitization will result in the formation of a broader depletion region. This ultimately leads to a large resistance jump in the material during the response process. Also, the synergistic catalysis effect of AuPd bimetallic nanoparticles can reduce the activation energy between the substances, resulting in an accelerated reaction rate between acetone and reactive oxygen species and ultimately greatly shortening the ZnO gas sensor’s response and recovery time [35,57]. 

## 6. Conclusions

Zinc oxide nanorods are produced using a straightforward hydrothermal method, while Au, Pd and Au-Pd are deposited onto the nanorods via an in situ reduction process. Owing to the larger surface area of ZnO nanorods, the synergistic effect of AuPd bimetals and the chemical and electronic sensitization effect of noble metals, the ZnO gas sensor modified with Au-Pd exhibits a higher response, a quicker response and recovery time (7 s/56 s) and better selectivity towards 50 ppm acetone. Moreover, the Au-Pd/ZnO gas sensor can still maintain good performance after one month. As a novel gas-sensing material, AuPd-modified ZnO nanorods provide a new way of detecting acetone in the atmosphere.

## Figures and Tables

**Table 1:** Gas sensitivity of different materials to acetone.

Material	T (°C)	C (ppm)	Response	t_res_ (s)	Ref.
SnO_2_/Au-doped In_2_O_3_ nanofibers	280	100	14	2	[40]
ZnO nanorods	219	100	12.9	13	[41]
ZnO-rGO nanofibers	200	200	4	36	[42]
Au-SnO_2_ nanosheets	240	100	18.18	5	[43]
Pt-CuFe_2_O_4_ nanotubes	320	100	17.5	/	[44]
Ag/ZnO nanoneedles	370	100	18	10	[45]
Au-Pd/ZnO nanorods	225	50	14	7	This work

## Data Availability

The data of this research are available upon request to the corresponding author.

## Supplementary Materials

The following supporting information can be downloaded at: https://www.mdpi.com/article/10.3390/s24072110/s1, Figure S1: Repeatability of Au-Pd/ZnO to 50 ppm acetone.
