# Supplementary material for: Enhanced Acetone Sensing Properties Based on Au-Pd Decorated ZnO Nanorod Gas Sensor
Source: Sensors (Basel). 2024 Mar 26;24(7):2110. doi: 10.3390/s24072110 (PMC11014327; doi:10.3390/s24072110)
Supplement: Supplementary file 1 [file sensors-24-02110-s001.zip › supplementary files.pdf]

Enhanced Acetone Sensing Properties Based on Au-Pd Decorated ZnO Nanorod Gas Sensor

Yinfeng Shen [1], Yiping Liu [1], Chao Fan [2], Qudong Wang [3], Ming Li [1], Zhi Yang [2,*] and Liming Gao[1,*]

1 State Key Laboratory of Metal Matrix Composites, School of Material Science and Engineering, Shanghai Jiao Tong University, Shanghai 200240, China
2 Key Laboratory of Thin Film and Microfabrication (Ministry of Education), Department of Micro/Nano Electronics, School of Electronic Information and Electrical Engineering, Shanghai Jiao Tong University, Shanghai 200240, China
3 National Engineering Research Center of Light Alloy Net Forming, State Key Laboratory of Metal Matrix Composites, School of Materials Science and Engineering, Shanghai Jiao Tong University, Shanghai 200240, China
* Correspondence: zhiyang@sjtu.edu.cn (Z.Y.); liming.gao@sjtu.edu.cn (L.G.)

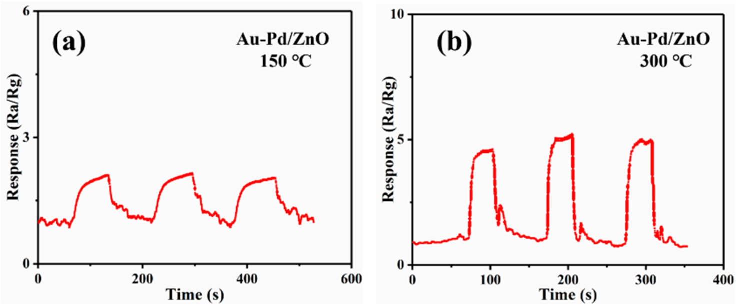

Figure S1 Repeatability of Au-Pd/ZnO to 50 ppm acetone.
